# Lights, Camera, and Action: Vertebrate Skin Sets the Stage for Immune Cell Interaction with Arthropod-Vectored Pathogens

**DOI:** 10.3389/fimmu.2013.00286

**Published:** 2013-09-17

**Authors:** Shu Zhen Chong, Maximilien Evrard, Lai Guan Ng

**Affiliations:** ^1^Functional Immune Imaging, Singapore Immunology Network (SIgN), Agency for Science, Technology and Research (A*STAR), Biopolis, Singapore; ^2^School of Biological Sciences, Nanyang Technological University (NTU), Singapore

**Keywords:** skin immunity, intravital imaging, host-pathogen interactions, skin imaging, two-photon microscopy

## Abstract

Despite increasing studies targeted at host-pathogen interactions, vector-borne diseases remain one of the largest economic health burdens worldwide. Such diseases are vectored by hematophagous arthropods that deposit pathogens into the vertebrate host’s skin during a blood meal. These pathogens spend a substantial amount of time in the skin that allows for interaction with cutaneous immune cells, suggesting a window of opportunity for development of vaccine strategies. In particular, the recent availability of intravital imaging approaches has provided further insights into immune cell behavior in living tissues. Here, we discuss how such intravital imaging studies have contributed to our knowledge of cutaneous immune cell behavior and specifically, toward pathogen and tissue trauma from the arthropod bite. We also suggest future imaging approaches that may aid in better understanding of the complex interplay between arthropod-vectored pathogens and cutaneous immunity that could lead to improved therapeutic strategies.

## Introduction

“Now, here, you see, it takes all the running you can do, to keep in the same place” – a statement made by the Red Queen to Alice in Lewis Carroll’s *Through the Looking Glass* in her explanation of the nature of Wonderland.

In 1973, Leigh Van Valen proposed the metaphor of an evolutionary arms race coined the Red Queen Hypothesis, which suggests that microbial pathogens and their host co-evolve continuously to maintain a state of balance (1). This continuous microbial challenge is believed to result in specialized immune cell subsets in the host (2) that reside in specific anatomical sites, which allows immune cells to defend against foreign pathogens yet maintain tolerance toward commensal flora (3, 4).

## The Stage and Actors: Vertebrate Skin, Immune Cells, and Arthropod Vectors

**Figure 1:**
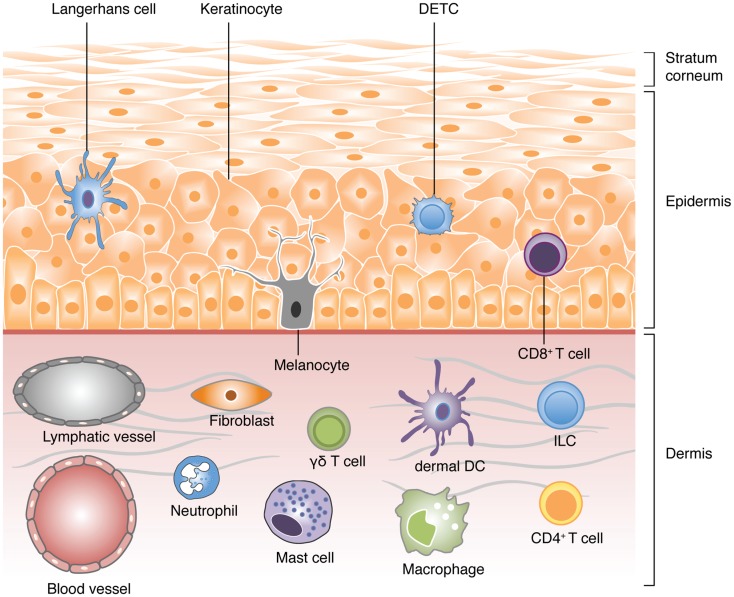
**A schematic view of the different cell types populating the skin**. Vertebrate skin is comprised of two major compartments: the epidermis and the dermis. The superficial part of the epidermis, known as the stratum corneum, is composed of dead keratinocytes and acts as a barrier. The epidermis is a dense and poorly vascularized region that comprises mainly of keratinocytes with few melanocytes. The major immune cells in this compartment include Langerhans cells (LCs), dendritic epidermal T-cells (DETC; a subset of γδ T-cells), and CD8 T-cells. The dermis is a highly vascularized region, rich in collagen, and elastin fibers, with low cell density. It comprises of fibroblasts, T-cells (CD4 αβ, and γδ), innate lymphoid cells (ILCs), dermal DCs (dDCs), macrophages, mast cells, and neutrophils (non-exhaustive list).

The skin serves as a primary example of an evolutionary adaptation of vertebrates. As the primary interface between the host’s body and environment, it provides a first line of defense against microbial pathogens and physical insults. Anatomically, the skin can be categorized into two distinct layers separated by a basement membrane: the dermis and the epidermis (Figure 1). The epidermis is a non-vascularized compartment consisting mainly of keratinocytes, which are critical in shaping and maintaining the immune response (5). Langerhans cells (LCs) and a subset of γδ T-cells found in mice, known as dendritic epidermal T-cells (DETCs), are the major immune cell types in the epidermis and are both characterized by their defined dendritic-like yet sessile behavior (6–8). In contrast to the epidermis, the highly vascularized dermis compartment bustles with activity and consists of a variety of immune cells including dermal dendritic cells (dDCs), mast cells, macrophages, neutrophils, and both αβ and γδT-cells. The majority of these cells display crawling or scouting behaviors and utilize extracellular matrix (ECM) fibers, such as collagen and elastin fibers, as scaffolds for their navigation (9–14). These cells may also enter and leave the dermis via blood and lymphatic vessels respectively through a series of highly coordinated events involving the use of integrins and chemokine gradients (1, 15–17).

Despite the skin serving as a significant barrier, a number of pathogens have evolved to bypass this barrier by hitch-hiking on an arthropod vector. Arthropods form a major group of disease vectors that include mosquitoes, sand flies, ticks, and mites. These hematophagous vectors allow pathogens to be deposited directly into the dermis during a blood meal. Once in the dermis, pathogens must evade the immune response to establish an infection or navigate their way toward the systemic circulation for successful dissemination (2, 18).

## The Camera: Dynamic Imaging *In vivo*

The interaction between arthropod vectors, the pathogen, and the host’s immune response is a complex and multifactorial event. A better understanding of these immune responses must thus be assessed dynamically *in vivo* to capture the full sequence of events. Fortunately, the relatively accessible nature of the skin for live imaging has provided us critical information on the fundamental behavior of immune cells (3, 4, 11, 12, 19–21). In recent years, the availability of the multiphoton confocal microscopy (MPCM) has allowed for deep tissue imaging at a lower potential of phototoxicity and photobleaching compared to conventional microscopy techniques. An additional advantage of this technology is the ability to generate second and third harmonic signals that identifies structural elements within tissues, such as collagen and elastin (5, 22–25). This allows improved behavioral analysis of immune cells and pathogens since their navigation within the dermis involves the use of such structural elements (6–8, 26). The accessibility of genetically modified mice that expresses fluorescent reporters using cell-type specific promoters, coupled with the disposal of fluorescent-tagged pathogens, has also enhanced the labeling and visualization of specific cells (9–14, 27). Such visualization is further aided by image analysis softwares that allow the tracking of individual cells in a three-dimensional volume over time. When such information provided through dynamic imaging *in vivo* is combined with other classical immunological techniques, such as flow cytometry or protein analysis, this results in better understanding of the functional consequences of observations made during imaging.

Despite rapid developments in intravital imaging, specific imaging studies involving the simultaneous interaction of arthropod-vectored pathogens and skin immune cells are still relatively limited. Nevertheless in this short review, we will focus on a handful of studies that have so far provided further insights into the dynamic interaction between immune cells and arthropod-vectored pathogens.

## Act 1: The Arthropod Bite

**Figure 2:**
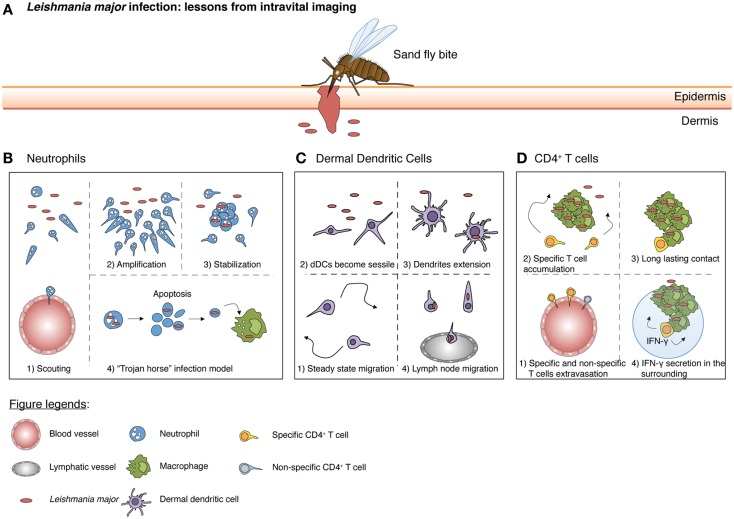
**A schematic summary from intravital imaging studies illustrating the responses initiated by different immune cells during *Leishmania major* infection**. **(A)** A sandfly bite creates a hemorrhagic pool and damages both the epidermis and dermis of the host. During a blood meal, parasites are then introduced into the dermis. **(B)** At the early stages, few scouting neutrophils are recruited at the lesion site (scouting phase) where parasites are localized. Subsequently, more neutrophils swarm toward the scouting neutrophils (amplification phase). Neutrophil clustering occurs, followed by stabilization (stabilization phase). Concurrently, neutrophils actively phagocytose the parasite. Neutrophils would eventually die by apoptosis and these infectious apoptotic bodies, containing *Leishmania major (L. major),* are scavenged by macrophages (“Trojan horse” model). **(C)** At steady state, dDCs patrol the dermal layer. However, upon parasites inoculation, dDCs become sessile and extend their dendrites, picking up parasites from neutrophil apoptotic bodies or capturing free *L. major* in the environment. Ultimately, dDCs migrate to the draining lymph nodes where they present antigens and initiate a T cell response. **(D)** Around 1 week after *L. major* deposition, antigen-specific CD4 T-cells are generated and can migrate to the site of infection. Both antigen-specific and non-specific CD4 T-cells can exit inflamed blood vessels, but only specific CD4 T-cells accumulate at the site of infection. Finally, through TCR/MHC-II interaction with infected macrophages, antigen-specific CD4 T-cells are able to produce IFN-γ. Of note, IFN-γ can act not only through cell contact, but also on cells in the surrounding vicinity via the “by-stander” effect for enhanced pathogen clearance.

Upon landing on the host’s skin, arthropods deposit pathogens directly into the dermis either through the probing of a proboscis or through mechanical wounding (Figure 2A). This act of injury to the epithelium and surrounding tissue is sufficient to activate and attract the first wave of immune cells even in the absence of a pathogen (28).

Although there are no specific arthropod-vectored imaging studies thus far that document the behavior of epidermal immune cells, DETCs have been demonstrated to be crucial in the response toward stress signals involving skin injury or trauma (29), which is a critical part of the arthropod bite. DETCs exhibit dendrites that were mostly oriented at the apical epidermis where they were immobilized at distal, cytoplasm-filled bulbous swellings (8). The basally orientated dendrites were highly mobile, exhibiting extending, and contracting movements as they reached into the dermis. DETCs recognize cells that are affected by stress through their TCRs and the natural killer (NK) cell family receptor, NKG2D (30). Following activation, DETCs respond to changes in epithelial cells by forming overt contacts with LCs (8, 31). Since LCs lack NKG2D, it is hypothesized that LCs rely on DETCs to receive specific information associated with NKG2D signaling (31). This then allows LCs to shape the outcome of immune responses by transmitting these information, along with signals they have obtained from cell trauma, such as damage associated molecular patterns (DAMPs) and cytokines release from damaged epithelial cells, to lymph nodes for priming of T-cells. Activation of DETCs also results in the retraction of their dendrites, which transforms them into round-looking cells. This morphological transformation is accompanied by cytokine production and is dependent on CD100 engagement on DETCs with keratinocyte-expressed plexin B2 (32). Similarly to DETCs, LCs survey their microenvironment by extending and retracting their dendrites whilst their cell body remained immobile (6, 7). This behavior was termed dSEARCH (dendritic surveillance extension and retraction cycling habitude) (9, 33). Upon activation, LCs were found to elongate their dendrites between keratinocyte tight junctions and access the epidermal layer directly beneath the stratum corneum (34). This process allows them to transport surface antigens into the cell body, where they subsequently accumulate in MHC-II rich compartments, without compromising the integrity of the epithelial layer (34, 35).

As the arthropod’s bite or proboscis reaches the dermal compartment of the skin, the innate immune system is activated and neutrophils are one of the first cells to be recruited into the site (36) (Figure 2B). Upon emigration from the blood vessels into the dermis, neutrophils display extremely coordinated chemotaxis and cluster formation that resemble the swarming of insects (37). Studies have shown that tissue damage inflicted as a result of the sand fly bite or by needle injection is sufficient to drive the early recruitment of neutrophils (38). Thus, it is likely that the first wave of neutrophil recruitment is mediated by the release of endogenous molecules during cellular injury, such as DAMPs, rather than pathogen-associated molecules (PAMPs) (39). We have previously shown that neutrophils patrol and scan normal tissue in the absence of inflammation (10). Upon the induction of sterile inflammation through laser burns, neutrophils crawl along the interstitium, relying on integrin-dependent signals toward the injury focus. This recruitment process occurs in a three-step cascade consisting of a (1) scouting phase (2) amplification phase and finally, (3) the stabilization of neutrophil clusters around the injury (10). The scouting phase relies on Gα-coupled receptor signaling while the amplification phase depends on the cyclic adenosine diphosphate ribose pathway. Recently, the lipid leukotriene B4 (LTB_4_) was also shown to mediate intercellular signal relay among neutrophils by amplifying local cell death signals to direct interstitial neutrophil recruitment during the amplification phase (40). This coordinated behavior of neutrophils was also observed during a sandfly bite with the presence of saliva components contributing to an amplification of neutrophil recruitment (41). Moreover, components of the salivary glands of *Lutzomyia intermedia* and *Lutzomyia longipalpis* was sufficient to result in the rapid influx of neutrophils (42, 43).

The recruitment of neutrophils is often closely associated with monocytes. Monocytes are heterogeneous, consisting of a Ly6C^hi^ population that is CCR2^hi^CX_3_CR1^lo^ and a Ly6C^lo^ population that is CCR2^lo^CX_3_CR1^hi^ (44). Ly6C^hi^ monocytes accumulate during inflammatory responses (45) while intravital imaging revealed that Ly6C^lo^ monocytes forage for micro-particles by crawling on the luminal side of the endothelium. This patrolling behavior relied on LFA-1 and CX_3_CR1 but was independent of chemokine signaling and direction of blood flow (46). Ly6C^lo^ monocytes can also be recruited relatively quickly during tissue injury and are one of the first few cells to produce TNF-α after extravasation (46). A recent study has suggested a phagocyte partnership between neutrophils and monocytes, whereby Ly6C^lo^ monocytes sense endothelial cell damage through TLR7 that results in the recruitment of neutrophils, leading to endothelial cell necrosis (47). Ly6C^lo^ monocytes then scavenge the resultant cell debris, which was hypothesized by the authors to limit excessive inflammation. In line with this finding, other studies have indicated that Ly6C^lo^ monocytes may be the first cells to alert neutrophils through chemokines and cytokines during tissue injury for neutrophil recruitment into the dermis (48). Subsequently, recruited neutrophils release granule contents that promote the extravasation of inflammatory Ly6C^hi^ monocytes, which then controls the further infiltration of neutrophils to prevent excessive tissue damage (40). Future intravital studies in the context of skin infections would thus be beneficial in dissecting the precise mechanisms behind this partnership.

## Act 2: The Immune Response Toward the Pathogen

Pathogens vectored by arthropods are often deposited directly into the dermis. Therefore, their recognition by the host must thus rely on dermal resident populations of DCs and macrophages. Using CD11c-promoter-driven expression of yellow fluorescent protein (YFP), we have previously showed that in comparison to the sessile LCs in the neighboring epidermis, dDCs actively crawled through the dermal interstitial space under homeostatic conditions (9) (Figure 2C). Their locomotive behavior depends on G-protein coupled receptors, suggesting that the displacement of dDCs involves chemokine or lipid mediators. Indeed, dDCs displayed intimate contact with the ECM and it is likely that chemoattractants are deposited along these structures. In line with this proposition, chemokines such as CCL21 was shown to have a highly charged C-terminal extension that binds glycoaminoglycans (GAGs), resulting in the immobilization of chemokines to ECM or cell surfaces (49). Upon sensing *Leishmania major*, lipopolysaccharide (LPS) or Bacillus Calmette–Guérin (BCG), dDCs arrested their migratory behavior (9). They also extended their long motile pseudopods and incorporated multiple parasites into intracellular vacuoles. However, the elaboration of dendrites did not occur in the presence of BCG and inert beads, suggesting a discriminatory behavior specifically toward parasites. Nevertheless, since both infected and uninfected dDCs were non-migratory at sites of *L. major* infection, their behavior is also likely to rely on the inflammatory environment rather than the uptake of parasites *per se*. Consequently, the arrest of dDCs near sites of infection/inflammation allows them to switch from surveillance to antigen uptake for efficient immune priming.

Dendritic cells have been shown to pick up antigens and travel through lymphatics to lymph nodes where they prime T-cells for activation. Interestingly, intravital imaging revealed that while effector T-cells entered the infected skin regardless of antigen-specificity, pathogen-specific T-cells preferentially accumulated in infected regions of *L. major* as they decreased their motility upon approach (50) (Figure 2D). However, antigen recognition by CD4 T-cells was not sufficient to establish stable contacts with every infected cell, as a substantial number of these T-cells were oblivious, thereby failing to engage infected cells. The accumulation of CD4 T-cells at infected sites was also disproportionately distributed, as not all sites were accessible to migrating T-cells. Nevertheless, this limited number of stable contacts was sufficient to create a “by-stander effect” that results in a gradient of effector signals (51). As such, pathogen control could be achieved within a 80-μm radius around the site of T cell-APC interactions. The “by-stander effector” activity of CD4 T-cells controlling intracellular pathogens is in contrast to cytotoxic CD8 T-cells, which have been shown to require individual contact with infected cells to trigger target cell apoptosis (52).

Neutrophils are recruited to the infectious site and are important for pathogen clearance through phagocytosis and release of microbicidal agents (36). They additionally mediate pathogen defense by releasing dense strands of DNA and proteins from within the cell body, a process known as neutrophil extracellular traps (NETs) (53). Using an *in vivo* model of *S. aureus* infection, it was demonstrated that the DNA-NET formation process (NETosis) in skin was TLR2 dependent and involved complement factor C3 mediated signaling (54). NETosis was shown to be crucial for limiting bacteria dissemination and was not observed during sterile injury, suggesting that this process was specifically directed against pathogens. Although *in vivo* imaging studies have yet to be conducted on arthropod-vectored pathogens, human neutrophils were shown to perform NETosis on *Leishmania* promastigotes (55), *Plasmodium falciparium* (56), and *Borrelia burgdoferi* (57). The precise mechanisms of NETosis in this context *in vivo* will thus be of interest in future imaging studies.

Macrophages are sessile immune cells that serve as obligatory phagocytes and final definitive hosts for replication of some arthropod-vectored pathogens (58). Upon pathogen adhesion to the cell membrane, usually with the tip or base of the flagellum, macrophages may exhibit “coiled phagocytosis” by wrapping their pseudopods around the pathogen before engulfing them (59). However, parasites such as *L. major* have evolved to hitch-hike onto the short-lived neutrophil (38), which acts as a “Trojan horse” and intermediate host in order for them to enter macrophages without cell activation (60). Similarly, the parasite may also adopt this “Trojan horse” strategy to evade the immune response by dDCs, as dDCs that captured infected neutrophils rather than the parasite itself resulted in an attenuated CD4 T-cell priming response (61). As such, the depletion of neutrophils during *L. major* infection resulted in decreased infection levels.

## Finale: Future Prospects

Despite increasing intravital imaging studies on host-pathogen interactions, only a handful are targeted specifically toward arthropod-vectored pathogens and cutaneous immunity through the natural route of infection. Currently, the most well defined arthropod-vectored pathogen in the context of cutaneous immunity is the *Leishmania* parasite. However, the interaction of skin immune cells with many other arthropod-vectored bacteria and viruses, despite being made available for imaging through fluorescent tagging, remain poorly described. In particular, the majority of *Plasmodium* parasite studies are performed in the liver, as it is the site of transformation from sporozoites into merozoites (62). However, the *Plasmodium* parasite was recently shown to linger in the dermis for an unexpected longer period of time and may even be the final destination for differentiation into persistent merozoites (63). Hence, these findings advocate for an urgent need toward more intravital imaging studies to be performed at the bite site in order to visualize the pathogen’s interaction with immune cells for design of potential vaccine strategies.

Additionally, due to the complex interaction of immune cells in response to pathogen, there is a substantial need to utilize transgenic mice that possess more than one fluorescent cell subset. For example, both monocytes and neutrophils are known to congregate at the locus of the sandfly bite (64) and such close interactions may influence not only the reciprocal immune cell behavior but also the outcome of *Leishmania* parasite behavior. Nevertheless, it remains challenging to simultaneously define a number of immune subsets without considerable overlap in fluorophores. However, by taking a cue out of the “brainbow” mouse model that allows for the differentiation of up to 90 colors in neurons (65), future approaches may allow for improved cell tracking of individual immune subsets. Therefore, development in these areas, coupled by the need for functional information to be simultaneously derived from imaging studies, would provide us with a much in-depth analysis and a global understanding of host-pathogen interactions.

## Conflict of Interest Statement

The authors declare that the research was conducted in the absence of any commercial or financial relationships that could be construed as a potential conflict of interest.
